# Psychological distress increases the risk of falling into poverty amongst older Australians: the overlooked costs-of-illness

**DOI:** 10.1186/s40359-018-0230-7

**Published:** 2018-04-17

**Authors:** Emily J. Callander, Deborah J. Schofield

**Affiliations:** 1 Australian Institute of Tropical Health and Medicine, James Cook University, Building 48, Douglas Campus, Townsville, QLD 4811 Australia; 2 Faculty of Pharmacy, The University of Sydney, Sydney, Australia

**Keywords:** Income, K10, Longitudinal analysis, Poverty, SF36

## Abstract

**Background:**

This paper aimed to identify whether high psychological distress is associated with an increased risk of income and multidimensional poverty amongst older adults in Australia.

**Methods:**

We undertook longitudinal analysis of the nationally representative Household Income and Labour Dynamics in Australian (HILDA) survey using modified Poisson regression models to estimate the relative risk of falling into income poverty and multidimensional poverty between 2010 and 2012 for males and females, adjusting for age, employment status, place of residence, marital status and housing tenure; and Population Attributable Risk methodology to estimate the proportion of poverty directly attributable to psychological distress, measured by the Kessler 10 scale.

**Results:**

For males, having high psychological distress increased the risk of falling into income poverty by 1.68 (95% CI: 1.02 to 2.75) and the risk of falling into multidimensional poverty by 3.40 (95% CI: 1.91 to 6.04). For females, there was no significant difference in the risk of falling into income poverty between those with high and low psychological distress (*p* = 0.1008), however having high psychological distress increased the risk of falling into multidimensional poverty by 2.15 (95% CI: 1.30 to 3.55). Between 2009 and 2012, 8.0% of income poverty cases for people aged 65 and over (95% CI: 7.8% to 8.4%), and 19.5% of multidimensional poverty cases for people aged 65 and over (95% CI: 19.2% to 19.9%) can be attributed to high psychological distress.

**Conclusions:**

The elevated risk of falling into income and multidimensional poverty has been an overlooked cost of poor mental health.

## Key points

What is already known:

1. Low income and poverty are risk factors for depression;
2. Depression has been identified as a risk factor for income poverty in working aged Australians;
3. Multidimensional measures of poverty look multiple aspects of people’s lives, not just income, and may pick up changes in living standards for older people who are no longer working.

What this study adds:

1. Older males who have high levels of psychological distress have an increased risk of falling into multidimensional poverty and income poverty;
2. Older females who have high levels of psychological distress have an increased risk of falling into multidimensional poverty but not income poverty;
3. Between 2009 and 2012, 8% of income poverty cases for people aged 65 and over, and 19.5% of multidimensional poverty cases for people aged 65 and over can be attributed to high psychological distress.

## Background

As the population of many countries age, an increasing proportion of the global population will be in their more advanced years [34]. As such, increasing attention is being paid to the wellbeing and living standards of older people [19, 22, 35, 53, 54]. Australia is no exception, with 25% of the population expected to be aged over 65 by 2044–45 [40].

Considerable attention has been paid to the macroeconomic impacts of an older population within Australia – increased health care costs, and welfare payments, and reduced productivity from a lower aged-dependency ratio (the proportion of working aged people to the proportion of people of retirement age) [50]. At the individual level, multiple studies have investigated the living standards of older people, which have included the assessment of health, income and other aspects of living standards [4, 5, 38]. One study by McRae et al., also explored the impact of healthcare costs on the living standards of older people within Australia, concluding that 12% faced catastrophic healthcare costs [33]. Older people with depression also faced the third highest annual costs for health care. This highlights growing interest in the health - living standards nexus, including mental health issues, amongst older Australians, and how this affects the well-being of a growing proportion of the population.

There is a vast body of literature that demonstrates the association between mental illness and lower socio-economic status in people of all ages [30, 31, 39, 41]. This literature, however, assesses the impact of income poverty on later mental health status, rather than the inverse relationship - the impact of mental illness on poverty. The direction of the impact is important for assessing points for potential intervention to ensure that older people are not beset by both poor mental health and poverty.

The limited literature assessing this inverse relationship, found that 57% of people aged 45 to 64 with depression were not in the labour force, and that these people had a weekly income 73% lower than people who were employed [42–45]. Butterworth et al. looked specifically at older workers and found that those who had retired early were more likely to have mental disorders than those in the labour force, particularly amongst men [7] and similar results were also reported by Gill et al. [24]. In a more recent study Butterworth et al. used longitudinal data of all ages to show that mental health status predicted future unemployment [8] and Kiely and Butterworth [29] used longitudinal data to show that mental health predicted receiving welfare payments [29]. However, these studies focus on the impact that mental ill health has upon unemployment or income and given that older people beyond the age of 65 have low rates of labour force participation these studies of lost earnings or the impacts on employment are likely to be insensitive to the full impact of mental illness on the living standards of people beyond the traditional retirement age. This paper aims to determine whether having a high level of psychological distress increases an older individual’s risk of falling into income poverty or multidimensional poverty and how many additional people fall into poverty as a result of high psychological distress,

## Methods

### Dataset sampling and weighting

This is a longitudinal study utilising the Household Income and Labour Dynamics in Australia (HILDA) Survey focusing on the Australian population aged 65 years and over in 2009. The HILDA survey is a longitudinal survey of private Australian households conducted annually since 2001. The data are nationally representative of the Australian population living in private dwellings and aged 15 years and over. The survey sampling unit for Wave 1 was the household, with all members of the household being part of the sample that would be followed for the life of the survey. The reference population for Wave 1 was all members of private dwellings in Australia, except overseas residents, including diplomatic personnel, in Australia; residents of institutions such as hospitals, military and police barracks, correctional institutions and monasteries and non-private dwellings such as hotels; and people living in very remote sparsely populated areas. Household sampling was conducted in a three-stage approach. Initially, 488 Census Collection Districts (each containing 200 to 250 households) were selected, within each district 22 to 34 dwellings were then selected, and finally up to three households within each dwelling were selected to be part of the sample [49].

There were 1516 records aged 65 and over on Wave 9 (2009), 111 records were excluded as they did not complete the Kessler 10 questionnaire or did not complete the entire self-completed questionnaire in Wave 9, and 535 records were excluded as they were already in income poverty in 2009, leaving a total sample of 870.

The initial household cross-sectional weights in Wave 1 (upon which the weights in subsequent waves are dependent) were derived from the probability of selecting the household and were calibrated so that the weighted estimates match known benchmarks for the number of adults by the number of children and state by part of state. The person-level weights were based on the household weights and then calibrated so that person weights match known benchmarks for sex by age, state by part of state, state by labour force status, marital status and household composition. Longitudinal weights adjust for attrition and were benchmarked against the characteristics of Wave 1. For a detailed description of HILDA weighting see Watson [52]). This paper focused on the continuing person sample from Waves 9 to 12.

### Income, health, education and poverty measures

There is a wide body of research within the poverty measurement field that measures multiple aspects of people’s lives, not just income, in order to assess poverty status and measure standards of living [1, 48]. The impact of poor mental health on multidimensional poverty status as been documented for people of working age [13]; however, the impact on older members of society, whose poverty status may be less influenced by employment income has not been explored. This study uses a multidimensional poverty measure, the Freedom Poverty Measure [9, 10], developed specifically for the Australian population. It has been used in the past to assess the multidimensional poverty status of different sub-populations [10, 14–16]. To determine an individual’s multidimensional poverty status the Freedom Poverty Measure measures income, health status and education attainment. Those who are in multidimensional poverty are considered to be in income poverty and have at least one other form of disadvantage. Those in multidimensional poverty are in one of the following three groups:

1. Those who had poor health and were in income poverty,
2. Those with an insufficient level of education attainment and were in income poverty,
3. Those who had poor health, an insufficient level of education attainment and were in income poverty.

Income poverty was based upon total regular annual household income, which was composed of regular private income (wages and salary, business income, investment income, and private pensions and transfers), Australian government public transfers (government income support payments and other government payments, such as family or carer payments), other public payments such as scholarships, and foreign pensions. This total income was then equivalised for the number and age of household members using the OECD-modified equivalence scale [18]. The cut-off point for being in income poverty was having an equivalised annual income less than 50% of the median equivalised annual income for the Australian population of all ages.

Health status was measured using the Physical Component Summary (PCS) and Mental Component Summary (MCS) scores from the SF-36 health scale [27], which was available from the HILDA dataset. The PCS was used to measure physical health and MCS was used to measure mental health. Those with poor health had a PCS or MCS less than 75% of the average for their age and were calculated each year.

Education attainment was measured based upon a person’s highest level of education attainment. Having achieved Year 9 or lower was considered to be an insufficient level of education attainment. It has been estimated that 45% of people aged 65 years and over have Year 9 or less as their highest level of education attainment, and a much higher proportion of this education group are amongst the lowest income earners than people with higher levels of education [11].

### Kessler 10 (K-10)

The Kessler Psychological Distress Scale (K10) of non-specific psychological distress [28] is a 10 item questionnaire about anxiety and depression symptoms the respondent experienced in the previous 4 weeks. It was administered as a part of the self-completion component of the HILDA survey. The Kessler 10 survey has been shown to be highly effective in screening for serious mental disorders and was found to strongly discriminate between DSM-IV/SCID cases and non-cases [21, 28] within the Australian population. The K10 produced kappa and weighted kappa scores ranging from 0.42 to 0.74 [17].

The HILDA survey included the K10 in Waves 7, 9 and 11, and grouped responses into four categories: low (score range 10–15), moderate (score range 16–21), high (score range 22–29), or very high (score range 30–50) [49]. There are a number of different approaches taken to the categorisation of K10 scores [2]. The approach utilised in the HILDA survey is based upon the approach utilised by the Australian Bureau of Statistics [2]. Due to low sample numbers, the authors re-grouped this variable to combine those with high and very high psychological distress (referred to as those with ‘high psychological distress’) and those with low and moderate psychological distress (referred to as those with ‘low psychological distress’). Participants were grouped based upon their response in wave 9 only.

### Statistical analysis

Descriptive analysis was undertaken to identify the baseline characteristics in 2009 of those who were not currently in income poverty. Two binary variables were created that identified any individual who experienced 1) income poverty or 2) multidimensional poverty between 2009 and 2012. The incidence of income poverty and multidimensional poverty between 2010 and 2012 based on the level of psychological distress was calculated and modified Poisson regression models [55] were constructed to estimate the relative risk for falling into income poverty and multidimensional poverty between 2010 and 2012 based on psychological distress category. Those who had low psychological distress were used as the reference group and the models were adjusted for age, employment status in 2009, remoteness of the place of residence in 2009, marital status in 2009 and housing tenure in 2009.

The analysis was conducted separately for males and females. This was because of the known and well-established differences in healthcare outcomes, and employment participation while of working age for males and females within Australia [3].

Modified Poisson regression analysis is a Poisson regression with a robust error variance, described by Zou [55]. Poisson regression is generally regarded as an appropriate method of analysis for events with a low probability (such as poverty) when respondents are followed over time [55]. However, traditional Poisson regression produces conservative error estimates [20], and the modified methodology described by Zou [55] provides a way of overcoming this.

A sensitivity analysis was conducted to exclude the potential of the SF-36 MCS – a summary score of mental health – influencing the results. The correlation co-efficient between the K10 score and SF-36 mental health score was − 0.81, *p* < .0001. Rather than the ‘poor health’ component of the multidimensional poverty measure being defined as having an MCS or PCS score less than 75% of the mean score for the respondents age, the sensitivity analysis defined ‘poor health’ as having only a PCS score less than 75% of the mean score for the respondents age (i.e. the MCS score was excluded). The modified Poisson regression models to estimate the relative risk for falling into multidimensional poverty between 2010 and 2012 based on psychological distress category was then repeated. Those who had low psychological distress were used as the reference group and the models were adjusted for age, employment status in 2009, remoteness of the place of residence in 2009, marital status in 2009 and housing tenure in 2009. The analysis was again conducted separately for males and females.

In order to estimate the proportion of income poverty and multidimensional poverty cases attributable to high psychological distress, the percent of cases that would be prevented if high psychological distress was eliminated was estimated using the population attributable risk method (PAR) [47]. This is based on the relative risk of income poverty and multidimensional poverty for high psychological distress, adjusted for age, sex, employment status in 2009, remoteness of the place of residence in 2009, marital status in 2009 and housing tenure in 2009, and the prevalence for the combinations of each of these risk factors. The partial PAR was calculated using an SAS macro developed by Hertzman et al. [26].

All of the analysis was undertaken on weighted data using SAS V9.2. Statistical significance was set at a 5% level.

## Results

There were 69 records of individuals aged 65 and over in 2009 on the HILDA dataset who had high psychological distress in 2009 (102,400 people in the Australian population), and 801 records of individuals who had low psychological distress (representing 1,111,500 people in the population).

Table 1 shows the demographic and employment characteristics in the baseline year, 2009. Of those who had high psychological distress, a higher proportion were female (58%) and not in the labour force (91%) and a lower proportion were employed (9%), compared to those with low psychological distress (51% female, 79% not in the labour force and 20% employed). A higher proportion of those with high psychological distress lived in outer regional and remote Australia than those with low psychological distress.

**Table 1:** Baseline characteristics, Australian population aged 65 years and over who were not already in income poverty in 2009

Characteristic	Low psychological distress		High psychological distress	
	n	N (%)	n	N (%)
Age – mean (SD)	72.5 (6.3)		73.0 (6.2)	
Female sex – no (%)	412	568,100 (51%)	42	67,400 (58%)
Male sex – no (%)	27	543,400 (49%)	394	35,000 (42%)
Area				
Major City	479	692,800 (62%)	41	59,100 (58%)
Inner Regional Australia	224	294,700 (27%)	16	20,700 (20%)
Outer Regional Australia	87	107,400 (10%)	11	19,700 (19%)
Remote Australia	16	16,500 (1%)	1	2800 (3%)
Labour force status				
Employed	170	222,200 (20%)	6	9500 (9%)
Unemployed	3	11,100 (1%)	0	0
Not in the labour force (retired)	633	878,100 (79%)	63	93,000 (91%)
TOTAL	801	1,111,500	69	102,400

Table 2 shows that between 2009 and 2012, 30% of people with low psychological distress in 2009 fell into income poverty, as compared to 49% of people with high psychological distress in 2009. Table 3 also shows that 18% of people with low psychological distress fell into multidimensional poverty between 2009 and 2012, and 48% of people with high psychological distress fell into multidimensional poverty between 2009 and 2012. Most people with low psychological distress who fell into multidimensional poverty had low income and an insufficient level of education attainment, whereas the majority of people with high psychological distress had low income and poor health, or low income, poor health and an insufficient level of education attainment (Table 2).

**Table 2:** Proportion of people who fell into poverty between 2009 and 2012, Australian population aged 65 years and over who were not already in income poverty in 2009

	Low psychological distress (*n* = 801; *N* = 1,111,500)		High psychological distress (*n* = 69; *N* = 102,400)	
	n	N(%)	n	N(%)
Income Poverty	251	336,200 (30%)	35	50,300 (49%)
Multidimensional Poverty – total	143	195,700 (18%)	33	48,800 (48%)
Multidimensional poverty – low income and poor health	34	57,000 (5%)	10	19,400 (19%)
Multidimensional poverty – low income and insufficient education attainment	80	110,100 (10%)	8	11,500 (11%)
Multidimensional poverty – low income, poor health and insufficient education attainment	29	29,600 (3%)	15	17,400 (17%)

a = low or moderate psychological distress as the reference group; adjusted for age, sex, employment in 2007 and remoteness of residence in 2007

**Table 3:** Modified Poisson regression model of incidence of income poverty between 2009 and 2012, Australian population aged 65 years and over

	Males		Females	
	Estimate (95% CI)	*p*-value	Estimate (95% CI)	*p*-value
Intercept	−2.97 (−5.07, − 0.88),	0.0053	− 1.28 (− 3.30, 0.74)	0.2149
High psychological distress	0.52 (0.02, 1.01),	0.0397	0.39 (− 0.08, 0.87)	0.1008
Age – continuous	0.02 (− 0.01, 0.05)	0.1684	−0.002 (− 0.03, 0.03)	0.9018
Not in the labour force	0.29 (−0.18, 0.75)	0.2252	0.27 (−0.20, 0.73)	0.2582
Major city	−0.09 (− 0.35, 0.33)	0.9603	− 0.09 (− 0.39, 0. 21)	0.5512
Married	0.15 (− 0.30, 0.61)	0.5155	− 0.05 (− 0.40, 0.31)	0.8008
Own home	− 0.06 (− 0.59, 0.48)	0.8375	0.15 (− 0.40, 0.71)	0.5900
Adjusted relative risk				
High psychological distress VS Low psychological distress	1.68 (1.02, 2.75)	0.0397	1.48 (0.93–2.37)	0.1008

When disaggregated by sex, 51% of males with high psychological distress and 28% of males with low psychological distress fell into income poverty, and 48% of females with high psychological distress and 33% of females with low psychological distress fell into income poverty. 51% of males with high psychological distress and 14% of males with low psychological distress fell into multidimensional poverty, and 46% of females with high psychological distress and 21% of females with low psychological distress fell into multidimensional poverty.

After adjusting for age, employment in 2009, remoteness of residence in 2009, marital status in 2009 and housing tenure in 2009, males with high psychological distress had 1.68 times the risk of falling into income poverty between 2010 and 2012 (95% CI: 1.02–2.75) compared to males with low psychological distress (Table 3). There was no significant difference in the risk of falling into income poverty between females with high and low psychological distress (*p* = 0.1008) (Table 3).

Having high psychological distress also increased the risk of both males and females falling into multidimensional poverty between 2009 and 2012, after adjusting for age, employment in 2009, remoteness of residence in 2009, marital status in 2009 and housing tenure in 2009. Males with high psychological distress had 3.40 times the risk (95% CI: 1.91–6.04), and females with high psychological distress had 2.15 times the risk (95% CI: 1.30–3.55) of falling into multidimensional poverty compared to their counterparts with low psychological distress (Table 4).

**Table 4:** Modified Poisson regression model of incidence of multidimensional poverty between 2009 and 2012, Australian population aged 65 years and over

	Males		Females	
	Estimate (95% CI)	*p*-value	Estimate (95% CI)	*p*-value
Intercept	−2.65 (−5.10, −0.21)	0.0333	−1.23 (−3.86, 1.39)	0.3571
High psychological distress	1.22 (0.65, 1.80)	<.0001	0.76 (0.26, 1.27)	0.0030
Age – continuous	0.01 (−0.02, 0.05)	0.4914	−0.006 (− 0.04, 0.03)	0.7310
Not in the labour force	0.17 (−0.48, 0.82)	0.6050	0.26 (−0.34, 0.86)	0.3961
Major city	−0.14 (− 0.64, 0.36)	0.5809	− 0.24 (− 0.63, 0.14)	0.2125
Married	− 0.45 (− 0.99, 0.08)	0.0982	0.07 (− 0.40, 0.54)	0.7710
Own home	0.14 (− 0.65, 0.92)	0.7357	0.02 (− 0.56, 0.59)	0.9560
Adjusted relative risk				
High psychological distress VS Low psychological distress	3.40 (1.91–6.04)	<.0001	2.15 (1.30–3.55)	0.0030

The sensitivity analysis, where the SF-36 MCS was removed from the multidimensional poverty measure, shows that both males (RR: 2.32, 95% CI: 1.17–4.60) and females (RR: 2.34, 95% CI: 1.39–3.93) with high psychological distress still had a significantly higher risk of falling into multidimensional poverty compared to those with low psychological distress –after adjusting for age, employment in 2009, remoteness of residence in 2009, marital status in 2009 and housing tenure in 2009 (Table 5).

**Table 5:** Sensitivity analysis modified poisson regression model of incidence of multidimensional poverty between 2009 and 2012, Australian population aged 65 years and over

	Males		Females	
	Estimate (95% CI)	*p*-value	Estimate (95% CI)	*p*-value
Intercept	−3.06 (−5.87, −0.24)	0.0332	0.44 (−2.26, 3.14)	0.7491
High psychological distress	0.84 (0.16, 1.53)	0.0160	0.85 (0.33, 1.37)	0.0013
Age – continuous	0.02 (−0.02, 0.06)	0.3345	−0.03 (− 0.07, 0.003)	0.0760
Not in the labour force	0.10 (−0.59, 0.80)	0.7726	0.38 (−0.25, 1.02)	0.2383
Major city	−0.34 (− 0.88, 0.21)	0.2240	− 0.29 (− 0.70, 0.11)	0.1578
Married	− 0.46 (−1.04, 1.22)	0.1211	0.12 (− 0.61, 0.36)	0.6185
Own home	0.12 (−0.73, 0.99)	0.7894	0.12 (−0.50, 0.74)	0.6959
Adjusted relative risk				
High psychological distress VS Low psychological distress	2.32 (1.17–4.60)	0.0160	2.34 (1.39–3.93)	0.0013

If all cases of high psychological distress in people aged 65 years and over in 2009 had been prevented than an estimated 8.0% of income poverty cases would have been avoided between 2010 and 2012 (95% CI: 7.8% to 8.4%), and an estimated 19.5% of multidimensional poverty cases would have been avoided between 2010 and 2012 (95% CI: 19.2% to 19.9%).

## Discussion

The results of this paper have shown that having high psychological distress increases the risk of older males falling into income poverty compared to those with only low psychological distress; however, there was no significant difference in the risk for older females between those with high and low levels of psychological distress. This is in line with the results of previous studies, which have found that while older males are more likely to retire early after developing mental disorders, the relationship was less pronounced for females [7, 24]. To date, no studies have specifically sought to use longitudinal data to document whether mental illness is a risk factor for income poverty.

In addition to showing the higher risk of income poverty, this study goes further by using a multidimensional measure of poverty, which captures a broader spectrum of factors that influence living standards. The results have shown that there is a significantly higher risk of both older males and females falling into multidimensional poverty amongst those with high psychological distress. The living standards of those in multidimensional poverty are seen to be poorer than those who are in income poverty but have no further forms of disadvantage, as those in multidimensional poverty not only have the burden of low income, but also have poor health or a relatively poor level of education attainment acting as barriers to improving their income, or indeed acting as a drain on their income (in the case of poor health [32]). As such, these findings identify older adults with high psychological distress as being a key target population for policies to improve living standards of vulnerable populations. Similarly, interventions to prevent high levels of psychological distress developing in older adults may be seen to have the additional indirect benefits of preventing cases of poverty. This study has indicated a need for policy to consider the multi-faceted needs of older people within the population. Health care and income support are generally delivered in silos, with little recognition of how health influences economic status and how economic status influences health. A more holistic approach to people’s wellbeing may be required, with better communication between sectors.

Studies that have looked at the costs of mental illness in terms of lost income have generally focused on lower labour force participation rates as a driver of low income, both within Australian and internationally ([6, 7, 24, 25, 36]; D Schofield et al., 2011; [46, 51]). However, given the older age group lower labour force participation is likely to only be part of the reason for lower income reported in this study, due to the majority of older people with and without high psychological distress being out of the labour force. None-the-less those with low psychological distress did have a higher proportion of people in employment and so the analysis adjusted for labour force status.

The high risk of falling into multidimensional poverty, even after controlling for employment status, may be explained by older people with high psychological distress and low income and poor overall health accessing their savings or accumulated wealth stocks to pay for their health condition (or conditions). The results did show that a high proportion of people with high psychological distress who were in multidimensional poverty had low income and poor health, rather than just low income and a low level of education attainment. Individuals aged 45 to 64 in Australia who had depression or another mental illness were more likely to have significantly less wealth or none at all [42, 44, 45]. A recent study of the out-of-pocket medical costs faced by older Australians listed depression as the third most costly condition, only behind heart disease and cancer [32], and another study has shown that over 40% of Australians with depression skip health care due to the cost [12]. In addition to the potential for medical costs to reduce the amount of wealth held by older people with psychological distress, it is known that those with depression and other mental health problems are less likely to hold income producing assets such as investment properties and shares [44, 45]. Thus, even if developing high psychological distress did not result in a drawdown of total wealth, it may result in a change in wealth portfolio structure to safer and less management intensive assets, which also produce lower returns, hence negatively affecting income.

Even after removing the SF-36 MCS, which measures overall mental health, from the measure of multidimensional poverty, both males and females with high psychological distress were still more likely to be multidimensionally poor, having low income plus either poor overall health status or an insufficient level of education attainment. This is likely to be explained by the findings of other studies, which have shown that amongst older adults, depression does increase the risk of a decline in physical health [23, 37].

The key limitation of this study is that it is based on self-reported data. Firstly, the study relies on responses to the Kessler-10 survey instrument and does not measure clinically diagnosed psychological distress. The measure also only asks respondents about their experiences in the previous 4 weeks. Despite this, the K10 is a validated survey instrument shown to have good validity and reliability (as noted in the methodology section). Furthermore, cognitive ability at baseline is a further potential confounder that was not available on the dataset and thus not included in the analysis. It should also been noted that although the HILDA dataset is accompanied by population weights, and weighted results are reported, the sample was truncated, with those already in income poverty excluded. This may have affected the accuracy of the population weights.

## Conclusions

Overall the results of this study have shown that older adults with high psychological distress have a higher risk of falling into income poverty and multidimensional poverty. Nearly half of older adults with high psychological distress in 2009 would fall into income poverty and multidimensional poverty by 2012. Even after adjusting for potential confounders, older males with high psychological distress had 1.7 times the risk of falling into income poverty and 3.4 times the risk of falling into multidimensional poverty, and older females had 2.2 times the risk of falling into multidimensional poverty, than their counterparts with low physiological distress. To date, these additional costs of psychological distress have not been quantified.

## Abbreviations

HILDA: Household Income and Labour Dynamics in Australia
K10: Kessler-10
MCS: Mental Component Summary
PAR: Population attributable risk
PCS: Physical Component Summary
